# Supplementary material for: Dynamic transcriptomic profiles of zebrafish gills in response to zinc supplementation
Source: BMC Genomics. 2010 Oct 11;11:553. doi: 10.1186/1471-2164-11-553 (PMC3091702; doi:10.1186/1471-2164-11-553)
Supplement: Additional file 2 — Interactive Direct Interaction Network representing the molecular interactions between zinc, copper, iron, calcium and proteins encoded by transcripts changed by zinc supplementation. Mini web-site containing index.html and hyperlinked pages in subdirectory describing a Direct Interaction Network automatically generated based on curated interactions contained within the proprietary PathwayArchitect database. Ovals represent proteins and the circles symbolize metal ions. Objects are coloured by their abundance in zebrafish at the time-point they were significantly different from the control is a scale from -4 fold (dark green) to +4 fold (dark red). Where significant differences were found at more than one time-point, the colour overlay shows expression at the first instance. Dark blue squares denote 'binding', and light blue squares 'expression'; green squares stand for 'regulation', green diamonds for 'metabolism', and green circles for 'promoter binding'. Arrow heads indicate directionality of the interaction where annotated. All nodes and edges can be further interrogated by selecting the relative area of the image. [file 1471-2164-11-553-S2.zip › PathwayArchitect Zn xs DIN/509149.html]

# REGULATION:

|  |  |
| --- | --- |
| Type | REGULATION |
| Effect | Positive |


---

|  |  |
| --- | --- |
| Score | 0 |


---

|  |  |
| --- | --- |
| Reference Count | 4 |


---

|  |  |
| --- | --- |
| Mechanism | Unknown |


---

|  |  |
| --- | --- |
| Reference:0 || Sentence | "These elements share no apparent sequence motif and bind different trans-acting proteins; a member of the NF kappa B family binds to the HIV-1 enhancer, the heterodimer of Jun and Fos (AP-1) binds to the collagenase enhancer, and the serum response factors p67 and p62 bind to fos." |
| PMID | 2557547 |
| Year | 1989 |
| Species | Human |
| Journal | Mol Cell Biol |
| RefScore | 0 |
| Source | PArchNLP |
  |
|  |  |


---

|  |  |
| --- | --- |
 Reference:1 || Sentence | "TNF alpha, and possibly IL1, enhances collagenase and stromelysin gene expression by stimulating the AP-1 promoter sites of the genes." |
| PMID | 7809909 |
| Year | 1994 |
| Species | Human |
| Journal | Tohoku J Exp Med |
| RefScore | 3 |
| Source | PArchNLP |
  ||  |  |


---

|  |  |
| --- | --- |
 Reference:2 || Sentence | "Retinoic acid inhibits the enzyme collagenase by forming an inactive complex between the liganded nuclear retinoic acid receptors and c-Jun, a protein that is itself an activator of the collagenase gene." |
| PMID | 1331926 |
| Year | 1992 |
| Species | Human |
| Journal | Nutr Rev |
| RefScore | 2 |
| Source | PArchNLP |
  ||  |  |


---

|  |  |
| --- | --- |
 Reference:3 || Sentence | A role for AP-1 was confirmed by over-expression of c-Jun and c-fos in confluent MDA-MB-231 cells, showing with c-Jun increased MMP-2 (5-fold), MMP-3 (1.6-fold), and MMP-9 (160-fold) expression, as well as enhanced invasive potential, while TIMP-1 expression was down-regulated (2-fold) when compared to vector controls. |
| PMID | 15777790 |
| Year | 2005 |
| Species | Human |
| Journal | Exp Cell Res |
| RefScore | 1 |
| Source | PArchNLP |
  |


---

|  |  |
| --- | --- |
